# What influences emergency general surgeons' treatment preferences for patients requiring nutritional support? A discrete choice experiment

**DOI:** 10.1111/codi.70081

**Published:** 2025-04-15

**Authors:** Daniel L. Ashmore, Jenna L. Morgan, Timothy R. Wilson, Vanessa Halliday, Matthew J. Lee

**Affiliations:** ^1^ School of Medicine and Population Health Faculty of Health, University of Sheffield Sheffield UK; ^2^ Department of General Surgery Doncaster and Bassetlaw Teaching Hospitals NHS Foundation Trust Doncaster UK; ^3^ Institute of Applied Health Research College of Medical and Dental Sciences, University of Birmingham Birmingham UK

**Keywords:** decision making, emergency general surgery, nutritional support

## Abstract

**Aim:**

Identifying and managing malnourished emergency general surgery (EGS) patients can be difficult. There are many tools available, a range of barriers to overcome and variety of guidelines at a surgeon's disposal. This study aimed to determine the impact of key variables on surgeon preference to start nutritional support in EGS.

**Methods:**

A discrete choice experiment was used to determine the impact of six variables on surgeons' treatment preferences for commencing nutritional support in EGS. Twenty‐five hypothetical scenarios regarding a patient with adhesional small bowel obstruction were disseminated electronically. Binomial logistic regression was used to identify significant associations. Ethical approval was obtained (UREC 050436).

**Results:**

In all, 148 participants responded providing 3700 scenario responses. Completion rate was 52.1% (148/284) with an approximately even split of consultants and non‐consultants (50.7% vs. 49.3%) and intestinal failure (IF) experience (46.6% experienced vs. 53.4% not experienced). Consultants favoured starting nutritional support (77.7%; 1443/1875) more often than non‐consultants (71.8%; 1310/1825). Forming an anastomosis, hypoalbuminaemia, underweight (body mass index <18.5 kg/m^2^), unintentional weight loss (>10%), ≥5 days without oral intake until now and ≥5 days likely to be without oral intake from now were statistically more likely to be associated with treatment preference, but obesity (body mass index >30 kg/m^2^) was not. Overall, experience of IF (OR 1.093, 95% CI 0.732–1.631; *P* = 0.663) and seniority of surgeon (OR 0.711, 95% CI 0.473–1.068; *P* = 0.100) significantly influenced the results.

**Conclusions:**

There are many variables that impact the decision to start nutritional support in EGS, but seniority of the surgeon and IF experience do not.

**What does this paper add to the literature?**

Using discrete choice methodology, we confirm that many variables influence the decision to start nutritional support in emergency general surgery patients, although it is not significantly influenced by surgeon seniority or experience of intestinal failure. The number of days likely to be without oral intake influenced decision making the most.

## INTRODUCTION

Emergency general surgery (EGS) patients may be malnourished or can become so during their hospital admission [1]. Malnutrition is associated with poor surgical outcomes [2, 3, 4]. It can be considered a modifiable risk factor if appropriate and adequate nutritional support is initiated [1].

Previous work has shown that there are several approaches used by surgeons to identify malnourished EGS patients, and these patients may benefit from nutritional support [5, 6]. Guidelines exist regarding initiation and timing of nutritional support, but they lack consistency and focus on the elective setting [7, 8, 9]. The mantra that oral intake is preferred to enteral tube feeding, which is preferred to parenteral nutrition (PN), prevails. Nutritional support is recommended for malnourished patients or those at risk of malnutrition within 5 days of admission according to the National Institute of Health and Care Excellence (NICE), although no information is given regarding timing pre‐ or post‐surgery [7]. The European Society for Clinical Nutrition and Metabolism (ESPEN) recommend nutritional support in patients who will be unable to eat for >5 days perioperatively [8] or those who cannot maintain >50% recommended intake for >7 days. PN can be initiated as soon as possible if there is a contraindication for enteral tube feeding, such as in intestinal obstruction [8]. The American Society for Parenteral and Enteral Nutrition (ASPEN) recognise that starting PN preoperatively in EGS patients is unlikely to be possible and so shortening the postoperative interval of PN can be considered, but only if its duration is likely to be longer than 7 days [9]. Additionally, ESPEN and ASPEN recommend the initiation of ‘early enteral nutrition’ (within 24–48 h) in critically unwell patients [9, 10], although it could be delayed for 1 week if not high risk [11]. These differences in guidelines may contribute to the lack of consensus in provision of nutritional support regarding timing, preferred route of administration and total energy targets worldwide [12].

On recognising that a patient may be malnourished, surgeons may then refer the patient to a nutritional support team or dietitian for further assessment with a view to starting nutritional support [13]. However, it is unknown what drives surgeons' decision making to start nutritional support, and to what extent factors such as surgeon seniority or experience of intestinal failure (IF) influence the decision making process.

## AIMS

This study aimed to determine the impact of key variables on surgeons' decision making regarding starting nutritional support in the EGS setting. Secondary aims included identifying whether treatment preference was associated with surgeon seniority or experience of IF.

## METHODS

A discrete choice experiment (DCE) was developed to study the surgeon preferences to start nutritional support using hypothetical scenarios in experimental conditions.

The DCE was part of a larger study, which also included a survey examining surgical nutrition practice in EGS (to be published in due course). Both were delivered simultaneously to the same population to limit participant burden. Only the DCE is reported here, and the relevant questions pertaining to the DCE are provided (Appendix S1).

### Study design

A DCE is a type of stated‐preference method that uses survey methodology to elicit the preferences of participants to hypothetical scenarios [14, 15]. It assumes that treatment preference is dependent on certain variables that can be ascribed to patient scenarios [14, 16]. DCEs were derived from health economics [17, 18] but have been used in the healthcare setting including in relation to prostate cancer [19], breast cancer [20], major gastrointestinal surgery [21] and from a nutrition perspective [22]. However, the method has not been used in the context of nutrition in EGS. It can be helpful to understand decision making regarding treatment choices where there is uncertainty of best practice or individual preference is important in the decision making process.

### Variable selection

Six variables which may influence outcome to start nutritional support were derived from the relevant literature [1, 5, 6], informed by guidelines [7, 8, 9, 10, 23, 24, 25, 26] and drawn from expert opinion of the study team. A summary of the selection of variables and levels is provided (Table 1), with a brief reasoning for the inclusion below.

- Management/operation: The formation of an anastomosis versus a stoma has been linked with decision making around the degree of risk surgeons are prepared to take and whether to start nutritional support [27].
- Albumin: Hypoalbuminaemia is regularly used as a marker for malnutrition in both research [5] and clinical practice [6] despite guideline recommendations otherwise [7, 8, 10, 23, 24].
- Body mass index (BMI): BMI is widely accepted as a marker of nutritional status [26]. A BMI <18.5 kg/m^2^ defines malnutrition in UK and European guidelines [7, 8, 28]. A low BMI (<20 kg/m^2^ if <70 years, <22 kg/m^2^ if >70 years), combined with other measures, can also be criteria for being malnourished [24, 28]. ASPEN recognise that patients with extreme low or high BMIs may be malnourished [25].
- Unintentional weight loss: More than 10% weight loss in 3–6 months defines malnutrition in UK guidelines [7]. ESPEN criteria for malnutrition include weight loss of 10%–15% within 6 months [8], while ASPEN use a range of weight loss criteria for malnutrition [25]. Global Leadership in Malnutrition (GLIM) guideline criteria include more than 5% weight loss within the past 6 months or more than 10% beyond 6 months [24].
- Days without oral intake: NICE recognise patients are at risk of malnutrition if patients have eaten, or are likely to eat, little or nothing for 5 days [7]. ESPEN and ASPEN recommend the initiation of ‘early enteral nutrition’ (within 24–48 h) in critically unwell patients, although if not high risk it may be delayed for 5–7 days [9, 10, 11]. Other guidelines reference reduced energy intake [8, 9, 24, 25]. These were captured by ‘days without oral intake until now’ (representing the time between symptom onset and surgical review) and ‘days likely to be without oral intake from now’ (representing the time between surgical review into the future).

**TABLE 1:** Discrete choice variables and levels.

Variable	Level 1	Level 2	Level 3	Level 4
Management/operation	**Continue conservative management**	Adhesiolysis	SB resection + anastomosis (no stoma)	SB resection + stoma
Albumin, g/L	< 25	25–29.9	30–34.9	**≥35**
BMI, kg/m^2^	<18.5	**18.5–24.9**	25–29.9	≥30
Unintentional weight loss, % in 3–6 months	**0–4.9**	5–10	>10	—
Days without oral intake until now	**1–4**	5–6	≥7	—
Days likely to be without oral intake from now	**1–4**	5–6	≥7	—

*Note*: Reference value in bold.

Abbreviations: BMI, body mass index; SB, small bowel.

### DCE development

**FIGURE 1:**
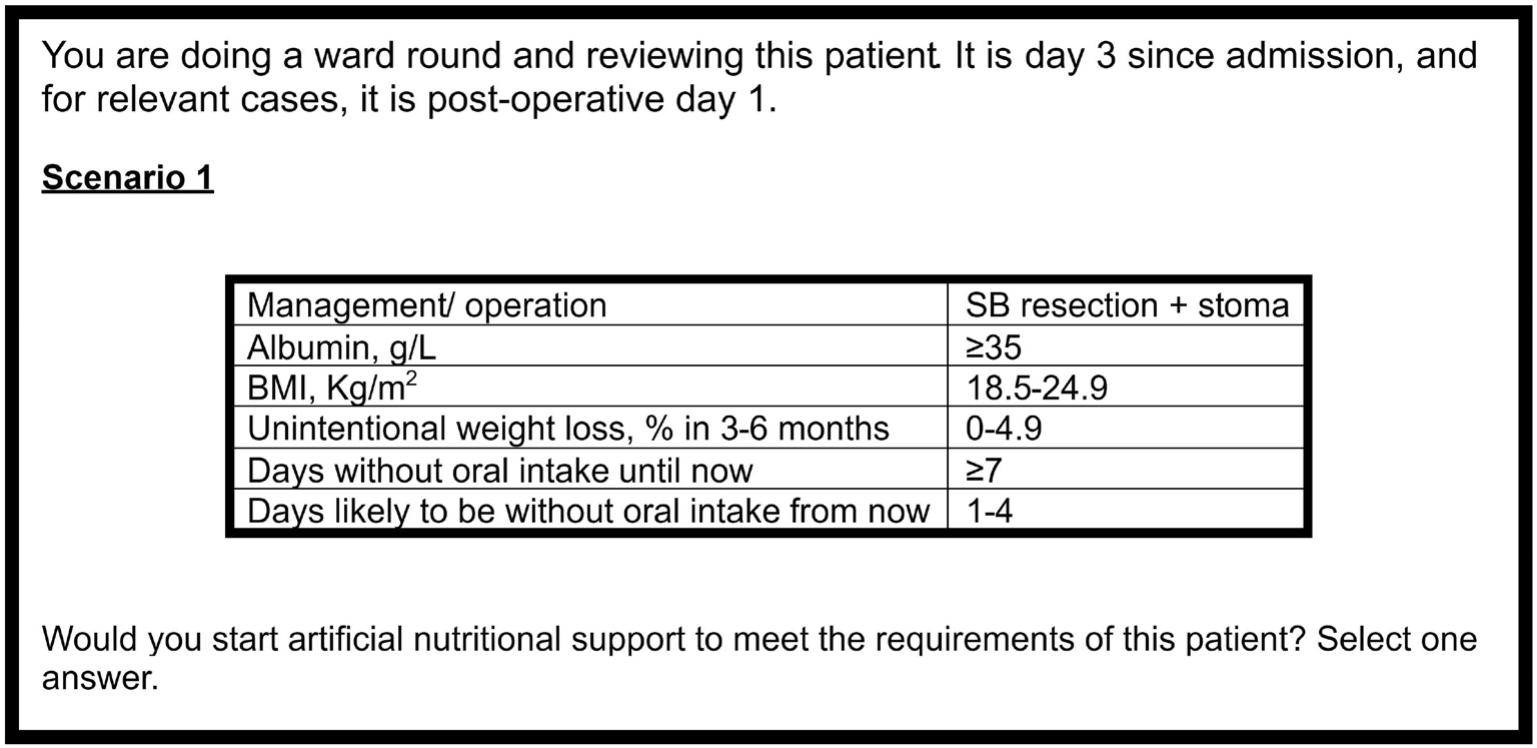
Scenario example.

Twenty‐five scenarios were randomly generated using SPSS v27 Orthoplan software out of a potential 1728 scenarios with an orthogonal design to minimise overlap [20, 29]. An example scenario is shown in Figure 1. The scenarios were presented to surgeons after introductory questions relating to current grade and intestinal experience. Scenario plausibility was checked with the expert panel, and no changes were made to those generated.

For each scenario, surgeons were asked to decide whether they ‘would start artificial nutritional support to meet the requirements of this patient?’ Surgeons could choose only ‘yes’ or ‘no’. Whilst it is recognised that surgeons may want to seek an opinion, this third option was not included as a pairwise design was considered to more closely reflect clinical practice [21]. To add clinical context to the study, all scenarios were prefixed with the same introductory stem (Figure 1). This stem allowed both conservative and operative management to be possible. Second, the days since admission represent typical time points when decisions around nutritional support might usually be made. The term ‘artificial nutritional support’ was used as this can encompass oral, enteral and parenteral options, which can be given in part or whole, alone or combined, to meet a patient's nutritional requirements. It was purposely chosen as it allowed surgeons to decide whether starting nutritional support would be beneficial without being prescriptive on the mode, yet also taking into account the complexity of decision making in such scenarios.

### Sample size

The minimum sample size needed for a DCE is complex. It depends on the statistical model used in the DCE analysis, an initial belief about the parameter values, the DCE design, as well as significance level (*a*) and statistical power level (1 – *b*) [30]. There were 4889 general surgeons in the National Health Service in March 2022, some of whom will be breast surgeons and do not participate in an EGS rota [31]. Given that the target population is relatively small, calculating a sample size was deemed unhelpful. Additionally, sample sizes a little over 100 are typically adequate to model preference data in discrete choice experiments [32], although it is possible with smaller samples [14].

### Ethical approval and informed consent

University of Sheffield ethical approval was obtained (UREC 050436). A Participant Information Sheet was emailed to potential participants which explained the purpose and duration of the study. Implied consent was given by participants clicking on a link in the invitation email, and subsequently on another link agreeing to continue on the welcome page of the study.

### Development and pre‐testing

The DCE was piloted among five consultant and non‐consultant general surgeons to ensure face and content validity. Feedback confirmed plausible scenarios and a mean time for completion of 10 min for the DCE section (although 15 min overall as it took 5 min for the survey section of the study). Fewer scenarios per page were recommended and this was addressed in the final design.

### Recruitment and survey administration

Surgeons working at registrar level or above were eligible to complete the DCE. This included surgeons in training and fellows post‐certificate of completion of training, as well as specialty and associate specialist (SAS) doctors, associate specialists and trust grade surgeons. Surgeons working below the level of a registrar were excluded.

The 25 scenarios were transformed into a survey format for online dissemination. The final study was publicised to members of the Association of Surgeons of Great Britain and Ireland (ASGBI), for which the study had received research support. Surgeons who had completed previous studies of a similar nature were also invited, as were members of general surgical trainee‐ and EGS‐specific groups on social media platforms. Responses were gathered using Qualtrics survey software, which also records the number of incomplete responses. Along with a click‐counting URL, this enabled a response rate to be calculated.

The study opened in December 2023 for 10 weeks. There were two additional reminders to complete it and no incentives were offered. Responses were anonymous, although participants were able to leave their email if they wished to be contacted in the future, be informed of findings or avoid reminder emails. Participants were able to review answers until submission.

### Analysis

Descriptive analyses were performed in IBM SPSS v27. Binomial logistic regression was performed to test for associations between treatment preferences and each of the variables, with responses clustered at participant level. *P* values <0.05 were considered significant. A similar analysis was performed for surgeons based on seniority (consultant vs. non‐consultant) and experience of IF, which was self‐reported and not defined. Not starting nutritional support was treated as the reference category.

## RESULTS

### Participant results

A total of 284 responses were recorded, of which 149 were completed. One response was excluded as the surgical trainee was not a specialty trainee or consultant. The completion rate was 52.1% (148/284). Half the surgeons were consultants (50.7%, 75/148) and almost half had experience of IF (46.6%, 69/148). Ten consultants (13.3%, 10/75) worked in an IF unit. All training regions in the UK were represented, with 64/148 of responses (43.2%) from Yorkshire and Humber. A summary of the included participants is shown in Table 2.

**TABLE 2:** Summary characteristics of the included participants.

Scenario	Responses, % (*n*)
Grade	
Consultant	50.7 (75)
Non‐consultant	49.3 (73)
Senior registrar (ST7‐8/post‐CCT Fellow)	14.9 (22)
Junior registrar (ST3‐6)	25.0 (37)
SAS doctor, associate specialist, trust grade	9.5 (14)
Consultants with IF experience	
Yes	57.3 (43)
No	42.7 (32)
Participants with IF experience	
Yes	46.6 (69)
No	53.4 (79)
Consultants working in an IFU	
Yes	13.3 (10)
No	86.7 (65)
Region of work	
Yorkshire and the Humber	43.2 (64)
North West (North West)	9.5 (14)
West Midlands	7.4 (11)
Kent, Surrey and Sussex	4.1 (6)
South West (Severn)	3.4 (5)
Wessex	3.4 (5)
Thames Valley	2.7 (4)
North West (Mersey)	2.7 (4)
Wales	2.7 (4)
South West (Peninsula)	2.7 (4)
North East	2.0 (3)
North West London	2.0 (3)
Northern Ireland	2.0 (3)
East of England	2.0 (3)
Ireland	2.0 (3)
Scotland (South East—Edinburgh)	1.4 (2)
East Midlands (South)	1.4 (2)
East Midlands (North)	1.4 (2)
Scotland (West—Glasgow)	0.7 (1)
South London (South West)	0.7 (1)
North Central and East London	0.7 (1)
South London (South East)	0.7 (1)
Scotland (North—Aberdeen)	0.7 (1)
Scotland (East—Dundee)	0.7 (1)

*Note*: Responses as % (*n*).

Abbreviations: CCT, certificate of completion of training; IF, intestinal failure; IFU, intestinal failure unit; SAS, specialty and associate specialist; ST, specialty trainee.

### Scenario results

**FIGURE 2:**
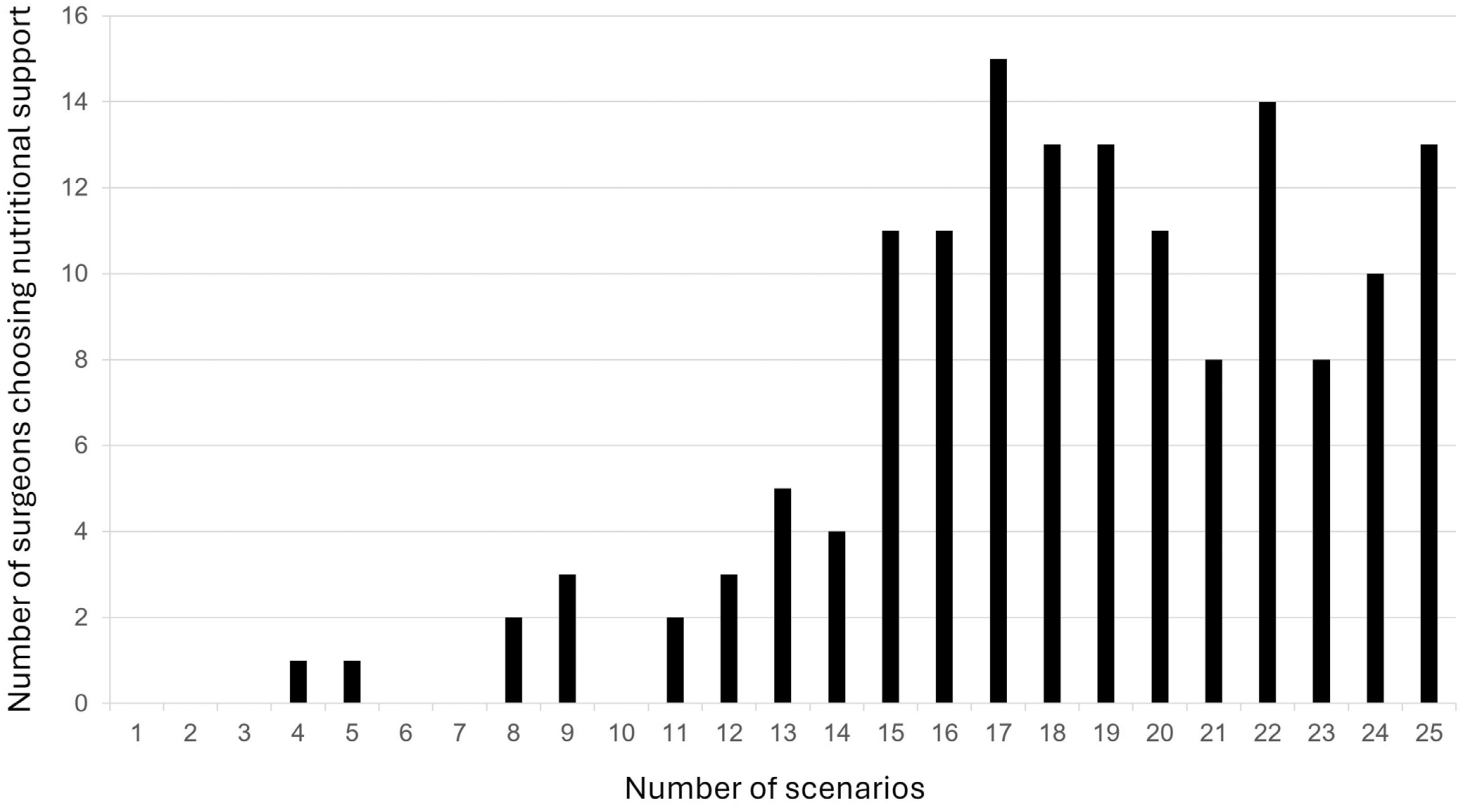
Preference for nutritional support.

The 148 surgeons answered 3700 scenarios (148 × 25); there were no missing answers. In 74.4% (2753/3700) of the scenarios surgeons preferred starting nutritional support, and in 25.6% of scenarios (947/3700) surgeons preferred not to start nutritional support. Overall, 91% (136/148) of surgeons favoured starting nutritional support in most scenarios (Figure 2), with nutritional support being favoured in 23 of the 25 scenarios (Table 3). However, there was treatment uncertainty for 14/25 scenarios, defined as less than 85% of surgeons choosing the same treatment preference (scenarios 1, 3, 4, 5, 8, 9, 11, 12, 13, 15, 19, 20, 21, 25). These included scenarios containing variables ‘1–4 days without oral intake until now’ (7/10 scenarios) and ‘1–4 days likely to be without oral intake from now’ (9/10 scenarios), and 7/10 scenarios (70%) containing 5%–10% unintentional weight loss.

**TABLE 3:** Results by scenario.

Scenario	Diagnosis	Albumin (g/L)	BMI (kg/m^2^)	Unintentional weight loss	DWOI until now	Days likely to be WOI from now	For nutritional support	Not for nutritional support
1	SB resection and stoma	≥35	18.5–24.9	0–4.9	≥7	1–4	60.8 (90)	39.2 (58)
2	SB resection and anastomosis	30–34.9	<18.5	0–4.9	1–4	≥7	95.9 (142)	4.1 (6)
3	Continue conservative management	30–34.9	25–29.9	0–4.9	≥7	1–4	75.0 (111)	25.0 (37)
4	SB resection and anastomosis	<25	≥30	0–4.9	5–6	1–4	69.6 (103)	30.4 (45)
5	Continue conservative management	<25	<18.5	0–4.9	1–4	1–4	58.8 (87)	41.2 (61)
6	Continue conservative management	30–34.9	18.5–24.9	>10	5–6	5–6	91.9 (136)	8.1 (12)
7	Continue conservative management	<25	≥30	0–4.9	5–6	5–6	86.5 (128)	13.5 (20)
8	Adhesiolysis	30–34.9	<18.5	5–10	5–6	1–4	64.9 (96)	35.1 (52)
9	SB resection and stoma	25–29.9	<18.5	0–4.9	1–4	5–6	76.4 (113)	23.6 (35)
10	Adhesiolysis	<25	<18.5	5–10	≥7	5–6	98.6 (146)	1.4 (2)
11	SB resection and anastomosis	≥35	25–29.9	5–10	1–4	5–6	62.2 (92)	37.8 (56)
12	SB resection and stoma	<25	<18.5	5–10	5–6	1–4	74.3 (110)	25.7 (38)
13	Continue conservative management	25–29.9	<18.5	>10	1–4	1–4	53.4 (79)	46.6 (69)
14	Continue conservative management	≥35	<18.5	0–4.9	5–6	5–6	87.8 (130)	12.2 (18)
15	SB resection and anastomosis	25–29.9	18.5–24.9	5–10	5–6	1–4	57.4 (85)	42.6 (63)
16	Adhesiolysis	<25	18.5–24.9	0–4.9	1–4	≥7	90.5 (134)	9.5 (14)
17	Adhesiolysis	≥35	≥30	>10	1–4	1–4	14.9 (22)	85.1 (126)
18	SB resection and anastomosis	<25	<18.5	>10	≥7	5–6	98.6 (146)	1.4 (2)
19	Continue conservative management	<25	18.5–24.9	5–10	1–4	5–6	78.4 (116)	21.6 (32)
20	SB resection and stoma	30–34.9	≥30	5–10	1–4	5–6	58.1 (86)	41.9 (62)
21	Continue conservative management	<25	25–29.9	5–10	1–4	1–4	29.1 (43)	70.9 (105)
22	Continue conservative management	≥35	<18.5	5–10	5–6	≥7	98.6 (146)	1.4 (2)
23	Continue conservative management	25–29.9	≥30	5–10	≥7	≥7	98.0 (145)	2.0 (3)
24	SB resection and stoma	<25	25–29.9	>10	5–6	≥7	99.3 (147)	0.7 (1)
25	Adhesiolysis	25–29.9	25–29.9	0–4.9	5–6	5–6	81.1 (120)	18.9 (28)

*Note*: *n* = 148 surgeons.

Abbreviations: BMI, body mass index; DWOI, days without oral intake; SB, small bowel; WOI, without oral intake.

### Influence of variables

**FIGURE 3:**
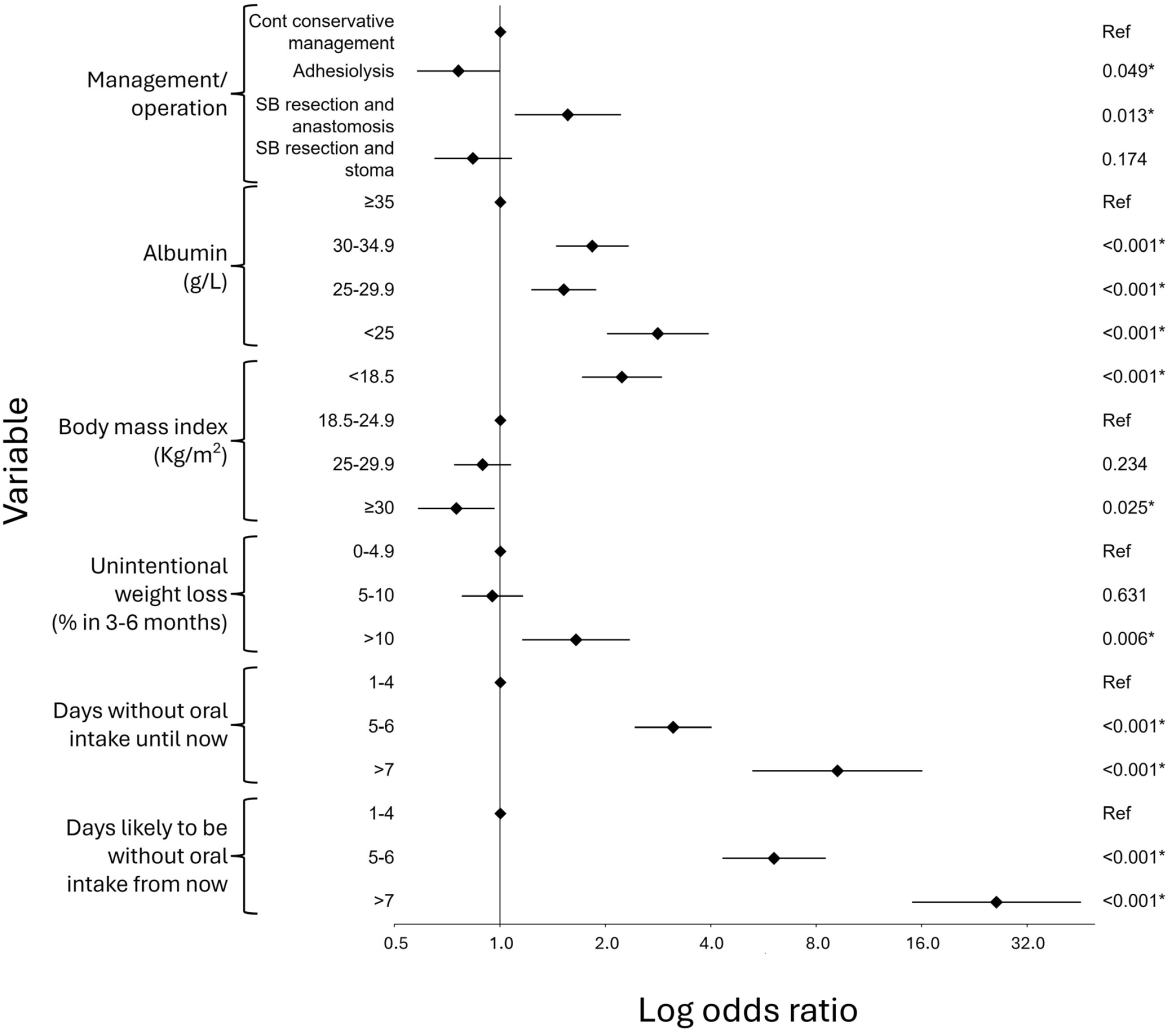
Forest plot of log odds ratios with 95% confidence intervals for each of the different attributes in the binary logistic regression analysis.

Three variables demonstrated an independently significant association with a preference for starting nutritional support at all levels on binominal logistic regression (Table 4, Figure 3). These included hypoalbuminaemia, ‘days without oral intake until now’ and ‘days likely to be without oral intake from now’. There was an increased preference for starting nutritional support for each successive number of days without oral intake (both ‘until now’ and ‘from now’).

**TABLE 4:** Influence of DCE variables over treatment choice (*n* = 148 surgeons).

Variable	Level	OR	95% CI (lower)	95% CI (upper)	*P* value
Management/operation	Continue conservative management	Ref	—	—	—
	Adhesiolysis	0.761	0.580	0.999	0.049^a^
	SB resection + anastomosis	1.563	1.102	2.216	0.013^a^
	SB resection + stoma	0.838	0.650	1.082	0.174
Albumin (g/L)	≥35	Ref	—	—	—
	30–34.9	1.837	1.447	2.331	<0.001^a^
	25–29.9	1.523	1.229	1.886	<0.001^a^
	<25	2.826	2.024	3.947	<0.001^a^
Body mass index (kg/m^2^)	18.5–24.9	Ref	—	—	—
	<18.5	2.232	1.715	2.906	<0.001^a^
	25–29.9	0.893	0.740	1.077	0.234
	≥30	0.751	0.584	0.965	0.025^a^
Unintentional weight loss (% in 3–6 months)	0–4.9	Ref	—	—	—
	5–10	0.952	0.778	1.165	0.631
	>10	1.651	1.159	2.351	0.006^a^
Days without oral intake until now	1–4	Ref	—	—	—
	5–6	3.126	2.428	4.024	<0.001^a^
	>7	9.200	5.262	16.084	<0.001^a^
Days likely to be without oral intake from now	1–4	Ref	—	—	—
	5–6	6.064	4.317	8.519	<0.001^a^
	>7	26.171	15.022	45.595	<0.001^a^

*Note*: *n* = 148 surgeons.

Abbreviations: CI, confidence interval; DCE, discrete choice experiment; OR, odds ratio; SB, small bowel.

^a^ Significant results.

Three variables offered a mixed picture. First, in comparison to the reference value of continuing conservative management, scenarios with a small bowel resection and anastomosis were significantly associated with preference for starting nutritional support. The contrary was true for adhesiolysis alone; it was significantly associated with a preference for not starting nutritional support. A small bowel resection and stoma was associated with a preference for not starting nutritional support, but this was not significant. Second, incremental increases in BMI were associated with a preference for not starting nutritional support. Consequently, scenarios which featured a BMI less than 18.5 kg/m^2^ were significantly associated with a preference for starting nutritional support, yet it was not for scenarios with a BMI more than 30 kg/m^2^. Finally, >10% unintentional weight loss in 3–6 months was associated with a preference to start nutritional support. This was not seen in scenarios with only 5%–10% weight loss.

### Influence of surgeon seniority

Whilst consultants favoured starting nutritional support (77.7%; 1443/1875) more often than non‐consultants (71.8%; 1310/1825), there was no significant association with surgeon seniority (OR 1.406, 95% CI 0.936–2.114; *P* = 0.100) (Table 5). However, there was a general trend for increasing preference for nutritional support as surgeons progressed through training (Table S1). There were only two scenarios where consultants preferred to start nutritional support and non‐consultants preferred not to (scenarios 13 and 15, Table S1). In nine scenarios, there was a greater than 5% difference in preference for nutritional support between consultants and non‐consultants (scenarios 5, 8, 11, 13, 14, 15, 17, 19, 21).

**TABLE 5:** Influence of surgeon seniority and IF experience over treatment choice.

Variable	Level	OR	95% CI (lower)	95% CI (upper)	*P* value
Seniority	Non‐consultant	Ref	—	—	—
	Consultant	1.406	0.936	2.114	0.100
IF experience	No experience	Ref	—	—	—
	Experience	1.093	0.732	1.631	0.663

*Note*: *n* = 148 surgeons.

Abbreviations: CI, confidence interval; OR, odds ratio.

### Influence of intestinal failure experience

Consultant surgeons working in an IF unit had a greater preference for starting nutritional support (80.8%; 202/250) than consultants who did not work in an IF unit (76.4%; 1241/1625) (Table S2). Also, surgeons with self‐reported IF experience (75.7%; 1305/1725) were marginally more in favour of starting nutritional support than surgeons without experience (73.3%; 1448/1975) (Table S3). However, experience of IF did not confer any statistical significance with starting nutritional support (OR 1.093, 95% CI 0.732–1.631; *P* = 0.663) (Table 5).

Of note, there were six scenarios with five or more percentage points difference (and up to almost 40 percentage points in one scenario) where surgeons working in an IF unit would initiate nutritional support but surgeons not at an IF unit would not (scenarios 5, 11, 16, 19, 20, 21, Table S2). In contrast, there was only one scenario (scenario 9) with more than five percentage points difference where surgeons at non‐IF units were more likely to initiate nutritional support compared to surgeons at IF units. A similar trend, but with a lower percentage point difference, is seen when comparing surgeons with IF experience to those without IF experience. Surgeons with IF experience would start nutritional support more often than surgeons without IF experience (scenarios 5, 11, 13, 15, 19, 20 and 21); and the converse is true for scenario 4 (Table S3).

## DISCUSSION

This study confirmed that many variables influence the decision to start nutritional support in EGS patients. However, decision making is not significantly influenced by surgeon seniority or experience of IF.

Starting nutritional support was significantly associated with ‘days without oral intake until now’ and ‘days likely to be without oral intake from now’. Obtaining information about oral intake relies on a thorough patient history and contemporaneous medical records. Of all variables, ‘days likely to be without oral intake from now’ influenced decision making the most. This suggests that surgeons form a strategic nutritional plan based on likely prognosis, although predicting this can be difficult and often nuanced. Clinically, patients may have been vomiting, have undergone a trial period of conservative management with bowel rest and had little/no oral intake, have a prolonged operation, occasionally remain intubated and ventilated postoperatively, develop an ileus or may be slow to ‘build up’ to normal diet. Taken together, ‘days without oral intake until now’ and ‘days likely to be without oral intake from now’, it is often the case for patients to have an extended period of time with little or no nutritional intake. Recording this time frame, recognising patients are not meeting their nutritional needs and developing a clear plan to correct this is required by surgeons. Nutritional status is a dynamic process, and patients may become malnourished during their hospital stay if not so on admission [1]. Repeated screening and assessments are required to identify it early and are recommended [7, 28]. However, it was not possible to capture these data without substantially changing the nature of the study.

Any degree of hypoalbuminaemia influenced the decision to start nutritional support; the more severe the hypoalbuminaemia, the more likely nutritional support was to be started. It is an independent prognostic marker of mortality [33, 34, 35]. Hypoalbuminaemia is common but is not directly related to nutritional status in the acute setting where it typically behaves as a negative acute phase marker [25,26]. Albumin was included as a key variable as we have previously shown that it remains regularly used as a nutrition marker in both research and clinical practice [5, 6], despite guidelines recommending otherwise [7, 8, 10, 23, 24]. While surgeons may have interpreted hypoalbuminaemia in this study from either perspective, the scenarios stated it was ‘day 3 since admission’ and clearly represented an acute presentation. Any hypoalbuminaemia was unlikely to be related to malnutrition and more likely to be related to another cause such as the degree of inflammation/sepsis or disease burden. As such, it is surprising that hypoalbuminaemia influenced decision making regarding starting nutritional support as was shown in this study. More work is required to educate surgeons of this and change practice.

BMI is a commonly used, albeit quite crude, measure of nutritional status. This study found that a BMI <18.5 kg/m^2^ positively influenced decision making with a view to starting nutritional support. In contrast, a BMI of 25–29.9 kg/m^2^ was less likely to receive nutritional support (though not significant), and a BMI >30 kg/m^2^ was significantly associated with not starting nutritional support. This may be related to how surgeons perceive what being malnourished means. It is easy to ‘see’ malnutrition in the form of underweight patients, whilst it can be hidden in overweight and obese patients [36, 37]. It may be considered by surgeons that overweight and obese patients can tolerate nutritional loss before it deleteriously impacts outcomes, and so nutritional support is not needed. However, there is an increasing overweight and obese population [38], and malnutrition may be missed in this cohort with potentially poor outcomes [39, 40, 41, 42].

A preference to initiate nutritional support was significantly associated with more than 10% unintentional weight loss in 3–6 months. This is reassuring as it is a key criterion to diagnose malnutrition in UK guidelines [7], and similar ranges are provided in other guidelines [8, 24, 25]. However, on closer inspection of scenarios where there was a difference in treatment preference between (1) consultant surgeons working at IF centres compared to those who do not (Table S2) and (2) all surgeons with IF experience compared to those without (Table S3), many of these scenarios involved a hypoalbuminaemia, a 5%–10% weight loss and 1–4 days without oral intake. A grounding in nutrition may afford surgeons the ability to observe for ‘soft markers’ of malnutrition, while those that have less exposure rely on ‘objective’ markers like albumin. This may explain why any level of hypoalbuminaemia influences decision making to a greater degree than 5%–10% weight loss (Figure 3).

Finally, the overall management and operation performed influenced whether surgeons would start nutritional support. An anastomosis meant surgeons were significantly more likely to start nutritional support, whilst they were less so if there was a stoma or there was adhesiolysis alone. The formation of an anastomosis has been linked with decision making around the degree of risk surgeons are prepared to take [27]. Surgeons may start nutritional support to ‘protect’ it, especially with a proximal anastomosis where the alternative of a high stoma is not a sensible option for the patient. Alternatively, surgeons may consider these patients will take longer to establish oral intake, and so nutritional support is likely to be required. Whilst forming an anastomosis and ‘days likely to be without oral intake from now’ may be linked, this study showed they independently increased the likelihood of starting nutritional support. However, patients who have undergone an adhesiolysis or had a stoma formed may also have had little or no nutritional intake for some time. Their nutritional recovery may be slower than anticipated, and nutritional support may be warranted in these patients too.

### Strengths

This DCE benefits from capturing 3700 surgeon responses to a single question, ‘would you start artificial nutritional support to meet the requirements of this patient?’ Participants across all grades of seniority, experience of IF and training region completed the study. The scenarios were based on six key variables drawn from the literature and guidelines, allowing us to understand the relative contribution of variables to decision making. Whilst each influenced decision making, caution should be exercised in using any in isolation when deciding if patients require nutritional support.

### Limitations

There are limitations to this study. Surgeons favoured starting nutritional support in 74.4% of scenarios. A national prospective cohort study which focused on the nutritional management of patients admitted to secondary care with acute small bowel obstruction found 35.9% of patients received a nutritional intervention in any form (oral, nasogastric/nasojejunal nutrition or PN) during their admission regardless of malnutrition risk [1]. Only a total of 14.8% of patients with small bowel obstruction were started on total PN [1]. The gap between surgeons favouring nutritional support in this study compared to that seen in clinical practice may be due to several factors. First, studies using survey methodology, including DCEs, will invariably suffer from selection bias. Nutrition is considered by some surgeons to be a ‘non‐surgical’ component of surgical management [43], and so only those with an interest in nutrition may have been inclined to participate. However, the design and development of the study were carefully considered to minimise this. As well as key variables being drawn from the literature, guidelines and an expert group, the study was piloted and received support from the ASGBI which assisted with recruiting participants from a broad population. Second, this difference may represent the ideal scenario versus the practical circumstances and environment that surgeons work within. Several barriers exist to identifying malnourished surgical patients in the acute setting [43], and these may not have been reflected in this study; nor were data collected about such. Finally, the data from the national audit of small bowel obstruction may not reflect the contemporary appetite of surgeons' preference for wider use of nutritional support and its increasing recognition as an important element in a patient's management [1].

Additionally, experience of IF had an open definition and was determined by participants, as was the mode of artificial nutritional support.

Hypothetical scenarios may lose some clinical context. Whilst steps were taken to mitigate this by providing an informative clinical stem, they do not capture the dynamic situation encountered on a day‐to‐day ward round. Further, surgeons may wish to start nutritional support but know that it may not be enough ‘to meet the requirements of the patient’. This could have led participants, particularly those with a nutrition interest, to answer erroneously (i.e., they would start nutritional support ordinarily but not at the level of meeting requirements, and so answer ‘no’ in one of our scenarios). The results from this study rebut this because surgeons with IF experience started nutritional support more often than those without IF experience. Additionally, the clinical stem was related to small bowel obstruction. Whilst this allowed the ‘management/operation’ variable to be investigated as a potential influencer of decision making around nutritional support, there is variation nationally and internationally in the management of this surgical emergency [44, 45]. For this reason, the study was limited to one nationality alone. However, it would be interesting to investigate whether the results are consistent internationally.

Whilst we selected six variables as the key drivers of decision making for surgeons based on literature, guidelines and expert panel, there is possibility that an important variable was not included, and the finer nuances of decision making cannot be captured in such a study. Arguably, ‘1–4 days without oral intake until now’ and ‘1–4 days likely to be without oral intake from now’ may mean a patient has no oral intake for 2–8 days—a very wide range. Other variables may also influence the decision making process such as access to nutritional support. However, splicing days without oral intake further or including more variables would dramatically increase the number of scenarios required and participant burden.

Finally, while surgeons may be proponents of starting nutritional support, decisions are rarely made in isolation. Other highly trained healthcare professionals including dietitians and gastroenterologists form part of the nutrition multidisciplinary team. Not all centres proclaim to have a nutrition support team [6, 46]. A comparative study investigating interprofessional differences in decision making may inform policy pathways.

### Implications for policy makers

First, this study has shown that several variables influence decision making to start nutritional support in EGS. Policy makers need to consolidate and ensure consistency across guidelines. Strategies to ensure implementation of these, perhaps by using national datasets such as the National Emergency Laparotomy Audit database or creating a new ‘small bowel obstruction database’, need to be considered. In particular, clarity needs to be provided for surgeons regarding use of albumin as a nutritional marker, as well as it being acknowledged that overweight and obese patients can be malnourished and may require nutritional support also. Surgeons should consider ‘soft’ nutrition indices such as small degrees of weight loss as part of their decision making process too.

Second, this DCE indicates an appetite for nutritional support which is not echoed clinically in national cohort studies [1]. Policy makers should investigate how access to nutritional support can be improved to meet this demand. Studies suggest this is limited by resources and funding [6].

### Implications for researchers

This study confirms that decision making by surgeons around nutritional support are made in the context of whether the patient has an anastomosis or a stoma following a bowel resection. These might be used as stratification or minimisation variables in future randomised trials of nutrition. Funding bodies may consider a commissioned call to inform nutritional support strategies in this setting.

## CONCLUSION

Variables derived from guidelines informed the development of hypothetical scenarios upon which surgeons were asked whether they would start nutritional support or not. This study showed that many variables influence the decision to give nutritional support in EGS, but surgeon seniority and IF experience do not. Days likely to be without oral intake influenced decision making the most.

## AUTHOR CONTRIBUTIONS

**Daniel L. Ashmore:** Conceptualization; data curation; formal analysis; investigation; methodology; project administration; visualization; writing – original draft; writing – review and editing. **Jenna L. Morgan:** Formal analysis; investigation; methodology; supervision; visualization; writing – review and editing. **Matthew J. Lee:** Methodology; writing – review and editing; visualization; supervision. **Timothy R. Wilson:** Supervision; visualization; writing – review and editing. **Vanessa Halliday:** Methodology; supervision; visualization; writing – review and editing.

## FUNDING INFORMATION

The University of Sheffield Institutional Open Access Fund funded the article processing charges. There were no other sources of funding.

## CONFLICT OF INTEREST STATEMENT

The authors declare no conflicts of interest.

## ETHICS STATEMENT

Ethical approval was received from the University of Sheffield, UK (UREC 050436).

## PREVIOUS COMMUNICATION

This study is not based on any previous communication to a society or meeting. It was presented as part of an oral presentation to the British Association of Parenteral and Enteral Nutrition 2024 annual conference.

## Supporting information


Data S1.

## Data Availability

Data can be made available on request.
