# Supplementary material for: What influences emergency general surgeons' treatment preferences for patients requiring nutritional support? A discrete choice experiment
Source: Colorectal Dis. 2025 Apr 15;27(4):e70081. doi: 10.1111/codi.70081 (PMC11997732; doi:10.1111/codi.70081)
Supplement: Supplementary file 1 — Data S1. [file CODI-27-0-s001.docx]

**What influences emergency general surgeons’ treatment preferences for patients requiring nutritional support? A discrete choice experiment**

Daniel L Ashmore^a,b^, Jenna L Morgan^a,b^, Timothy R Wilson^b^, Vanessa Halliday^a^, Matthew J Lee^c^

^a^School of Medicine and Population Health, Faculty of Health, University of Sheffield, Sheffield, UK

^b^Department of General Surgery, Doncaster and Bassetlaw Teaching Hospitals NHS Foundation Trust, Doncaster, UK

^c^Institute of Applied Health Research, College of Medical and Dental Sciences, University of Birmingham, Birmingham, UK

**Corresponding author**

Mr Daniel L Ashmore

School of Medicine and Population Health, Faculty of Health, University of Sheffield, Beech Hill Road, Sheffield, UK, S10 2RX

dlashmore1@sheffield.ac.uk

ORCID ID 0000-0002-4321-5613; Twitter @realdanashmore

**Supplementary Materials - Index**

| **Supplementary Appendices** |  |
| --- | --- |
| Appendix 1: The Discrete Choice Experiment questions and scenarios | *Page 3-30* |
| **Supplementary Figures and Tables** |  |
| Table S1: Preferences for nutritional support for each scenario according to surgeon seniority | *Page 31* |
| Table S2: Preferences for nutritional support for each scenario for consultants | *Page 32* |
| Table S3: Preferences for nutritional support for each scenario according to experience of intestinal failure | *Page 33* |

**Supplementary Appendices**

**Appendix 1**

**The Discrete Choice Experiment questions and scenarios**

Start of Block: Intro and thanks

Q1.1 Thank you for taking part in this study.

It takes approximately 15 minutes to complete. It is split into three sections. Questions about yourself. This will take 1 minute. Scenario-based questions. This will take 10 minutes. Questions about awareness, practice, barriers and training. This will take 3-5 minutes.  As a reminder, your responses will be anonymous. You will be able to leave this study and re-enter at a later time to finish it if required. 

By pressing next, you are consenting to continue with the study.

End of Block: Intro and thanks

Start of Block: Background

Q2.1 **Background**

| 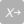 |
| --- |

Q2.2 What is your current grade? Please select one from the drop down list.

▼ Consultant (1) ... Other__________ (5)

Display This Question:

If What is your current grade? Please select one from the drop down list. = 5

Q2.3 If you selected 'other', please type your answer into the box here

________________________________________________________________

________________________________________________________________

Display This Question:

If What is your current grade? Please select one from the drop down list. = 1

| 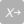 |
| --- |

Q2.4 Do you work in an intestinal failure unit?

- Yes (1)
- No (2)

| 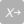 |
| --- |

Q2.5 Do you currently manage, or have previous experience of managing, patients on an intestinal failure unit?

- Yes (1)
- No (2)

| 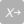 |
| --- |

Q2.6 What region do you currently work in?

▼ East Midlands (North) (1) ... Yorkshire & the Humber (24)

End of Block: Background

Start of Block: Introduction to scenarios

Q3.1 This section comprises a series of 25 clinical scenarios on which you are asked to make a hypothetical decision about whether to start artificial nutritional support to meet the requirements of the patient or not. In all cases, the patient has adhesional small bowel obstruction and has been in hospital for 3 days. In relevant scenarios, it is the first day following their surgery. You are doing a ward round and reviewing the patient.

We are interested in the importance that you place on six factors influencing your decision to start artificial nutritional support or not. The scenarios are presented in a short tabulated format, varying with the following aspects:

 **1. Management/ operation**
 *Divided into the following:* Continue conservative management (Mx) Adhesiolysis

Small bowel (SB) resection + stoma

Small bowel (SB) resection + anastomosis

  **2.  Albumin, g/L**
 *Divided into the following:* <25 25-29.9 30-34.9 ≥35

  **3. Body mass index, Kg/m2**
 *Divided into the following:*  <18.5 18.5-24.9 25-29.9 ≥30

  **4*.  Unintentional weight loss, % in 3-6 months***
 *Divided into the following:* 0-4.9 5-10 >10

  **5*.  Days without oral intake until now****.*
 *This is defined as the number of days the patient has eaten little or nothing between symptom onset and your review now.*
 *Divided into the following:* 1-4 5-6 ≥7

  **6*. Days likely to be without oral intake from now.***
 *This is defined as the number of days the patient will likely eat little or nothing from your review now.*
 *Divided into the following:* 1-4 5-6 ≥7

End of Block: Introduction to scenarios

Start of Block: Scenarios

| 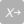 |
| --- |

Q4.1

You are doing a ward round and reviewing this patient. It is day 3 since admission, and for relevant cases, it is post-operative day 1.

**Scenario 1**

| Management/ operation | SB resection + stoma |
| --- | --- |
| Albumin, g/L | ≥35 |
| BMI, Kg/m^2^ | 18.5-24.9 |
| Unintentional weight loss, % in 3-6 months | 0-4.9 |
| Days without oral intake until now | ≥7 |
| Days likely to be without oral intake from now | 1-4 |

Would you start artificial nutritional support to meet the requirements of this patient? Select one answer.

- Yes (1)
- No (2)

| 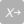 |
| --- |

Q4.2

You are doing a ward round and reviewing this patient. It is day 3 since admission, and for relevant cases, it is post-operative day 1.

**Scenario 2**

| Management/ operation | SB resection + anastomosis |
| --- | --- |
| Albumin, g/L | 30-34.9 |
| BMI, Kg/m^2^ | <18.5 |
| Unintentional weight loss, % in 3-6 months | 0-4.9 |
| Days without oral intake until now | 1-4 |
| Days likely to be without oral intake from now | ≥7 |

Would you start artificial nutritional support to meet the requirements of this patient? Select one answer.

- Yes (1)
- No (2)

| 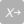 |
| --- |

Q4.3

You are doing a ward round and reviewing this patient. It is day 3 since admission, and for relevant cases, it is post-operative day 1.

**Scenario 3**

| Management/ operation | Continue conservative management |
| --- | --- |
| Albumin, g/L | 30-34.9 |
| BMI, Kg/m^2^ | 25-29.9 |
| Unintentional weight loss, % in 3-6 months | 0-4.9 |
| Days without oral intake until now | ≥7 |
| Days likely to be without oral intake from now | 1-4 |

Would you start artificial nutritional support to meet the requirements of this patient? Select one answer.

- Yes (1)
- No (2)

| 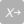 |
| --- |

Q4.4

You are doing a ward round and reviewing this patient. It is day 3 since admission, and for relevant cases, it is post-operative day 1.

**Scenario 4**

| Management/ operation | SB resection + anastomosis |
| --- | --- |
| Albumin, g/L | <25 |
| BMI, Kg/m^2^ | ≥30 |
| Unintentional weight loss, % in 3-6 months | 0-4.9 |
| Days without oral intake until now | 5-6 |
| Days likely to be without oral intake from now | 1-4 |

Would you start artificial nutritional support to meet the requirements of this patient? Select one answer.

- Yes (1)
- No (2)

| 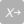 |
| --- |

Q4.5

You are doing a ward round and reviewing this patient. It is day 3 since admission, and for relevant cases, it is post-operative day 1.

**Scenario 5**

| Management/ operation | Continue conservative management |
| --- | --- |
| Albumin, g/L | <25 |
| BMI, Kg/m^2^ | <18.5 |
| Unintentional weight loss, % in 3-6 months | 0-4.9 |
| Days without oral intake until now | 1-4 |
| Days likely to be without oral intake from now | 1-4 |

Would you start artificial nutritional support to meet the requirements of this patient? Select one answer.

- Yes (1)
- No (2)

| Page Break |  |
| --- | --- |

| 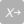 |
| --- |

Q4.6

You are doing a ward round and reviewing this patient. It is day 3 since admission, and for relevant cases, it is post-operative day 1.

**Scenario 6**

| Management/ operation | Continue conservative management |
| --- | --- |
| Albumin, g/L | 30-34.9 |
| BMI, Kg/m^2^ | 18.5-24.9 |
| Unintentional weight loss, % in 3-6 months | >10 |
| Days without oral intake until now | 5-6 |
| Days likely to be without oral intake from now | 5-6 |

Would you start artificial nutritional support to meet the requirements of this patient? Select one answer.

- Yes (1)
- No (2)

| 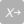 |
| --- |

Q4.7

You are doing a ward round and reviewing this patient. It is day 3 since admission, and for relevant cases, it is post-operative day 1.

**Scenario 7**

| Management/ operation | Continue conservative management |
| --- | --- |
| Albumin, g/L | <25 |
| BMI, Kg/m^2^ | ≥30 |
| Unintentional weight loss, % in 3-6 months | 0-4.9 |
| Days without oral intake until now | 5-6 |
| Days likely to be without oral intake from now | 5-6 |

Would you start artificial nutritional support to meet the requirements of this patient? Select one answer.

- Yes (1)
- No (2)

| 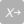 |
| --- |

Q4.8
 

You are doing a ward round and reviewing this patient. It is day 3 since admission, and for relevant cases, it is post-operative day 1.

**Scenario 8**

| Management/ operation | Adhesiolysis |
| --- | --- |
| Albumin, g/L | 30-24.9 |
| BMI, Kg/m^2^ | <18.5 |
| Unintentional weight loss, % in 3-6 months | 5-10 |
| Days without oral intake until now | 5-6 |
| Days likely to be without oral intake from now | 1-4 |

Would you start artificial nutritional support to meet the requirements of this patient? Select one answer.

- Yes (1)
- No (2)

| 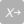 |
| --- |

Q4.9
 

You are doing a ward round and reviewing this patient. It is day 3 since admission, and for relevant cases, it is post-operative day 1.

**Scenario 9**

| Management/ operation | SB resection + stoma |
| --- | --- |
| Albumin, g/L | 25-29.9 |
| BMI, Kg/m^2^ | <18.5 |
| Unintentional weight loss, % in 3-6 months | 0-4.9 |
| Days without oral intake until now | 1-4 |
| Days likely to be without oral intake from now | 5-6 |

Would you start artificial nutritional support to meet the requirements of this patient? Select one answer.

- Yes (1)
- No (2)

| 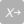 |
| --- |

Q4.10

You are doing a ward round and reviewing this patient. It is day 3 since admission, and for relevant cases, it is post-operative day 1.

**Scenario 10**

| Management/ operation | Adhesiolysis |
| --- | --- |
| Albumin, g/L | <25 |
| BMI, Kg/m^2^ | <18.5 |
| Unintentional weight loss, % in 3-6 months | 5-10 |
| Days without oral intake until now | ≥7 |
| Days likely to be without oral intake from now | 5-6 |

Would you start artificial nutritional support to meet the requirements of this patient? Select one answer.

- Yes (1)
- No (2)

| Page Break |  |
| --- | --- |

| 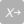 |
| --- |

Q4.11

You are doing a ward round and reviewing this patient. It is day 3 since admission, and for relevant cases, it is post-operative day 1.

**Scenario 11**

| Management/ operation | SB resection + anastomosis |
| --- | --- |
| Albumin, g/L | ≥35 |
| BMI, Kg/m^2^ | 25-29.9 |
| Unintentional weight loss, % in 3-6 months | 5-10 |
| Days without oral intake until now | 1-4 |
| Days likely to be without oral intake from now | 5-6 |

Would you start artificial nutritional support to meet the requirements of this patient? Select one answer.

- Yes (1)
- No (2)

| 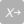 |
| --- |

Q4.12

You are doing a ward round and reviewing this patient. It is day 3 since admission, and for relevant cases, it is post-operative day 1.

**Scenario 12**

| Management/ operation | SB resection + stoma |
| --- | --- |
| Albumin, g/L | <25 |
| BMI, Kg/m^2^ | <18.5 |
| Unintentional weight loss, % in 3-6 months | 5-10 |
| Days without oral intake until now | 5-6 |
| Days likely to be without oral intake from now | 1-4 |

Would you start artificial nutritional support to meet the requirements of this patient? Select one answer.

- Yes (1)
- No (2)

| 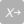 |
| --- |

Q4.13

You are doing a ward round and reviewing this patient. It is day 3 since admission, and for relevant cases, it is post-operative day 1.

**Scenario 13**

| Management/ operation | Continue conservative management |
| --- | --- |
| Albumin, g/L | 25-29.9 |
| BMI, Kg/m^2^ | <18.5 |
| Unintentional weight loss, % in 3-6 months | >10 |
| Days without oral intake until now | 1-4 |
| Days likely to be without oral intake from now | 1-4 |

Would you start artificial nutritional support to meet the requirements of this patient? Select one answer.

- Yes (1)
- No (2)

| 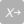 |
| --- |

Q4.14

You are doing a ward round and reviewing this patient. It is day 3 since admission, and for relevant cases, it is post-operative day 1.

**Scenario 14**

| Management/ operation | Continue conservative management |
| --- | --- |
| Albumin, g/L | ≥35 |
| BMI, Kg/m^2^ | <18.5 |
| Unintentional weight loss, % in 3-6 months | 0-4.9 |
| Days without oral intake until now | 5-6 |
| Days likely to be without oral intake from now | 5-6 |

Would you start artificial nutritional support to meet the requirements of this patient? Select one answer.

- Yes (1)
- No (2)

| 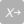 |
| --- |

Q4.15

You are doing a ward round and reviewing this patient. It is day 3 since admission, and for relevant cases, it is post-operative day 1.

**Scenario 15**

| Management/ operation | SB resection + anastomosis |
| --- | --- |
| Albumin, g/L | 25-29.9 |
| BMI, Kg/m^2^ | 18.5-24.9 |
| Unintentional weight loss, % in 3-6 months | 5-10 |
| Days without oral intake until now | 5-6 |
| Days likely to be without oral intake from now | 1-4 |

Would you start artificial nutritional support to meet the requirements of this patient? Select one answer.

- Yes (1)
- No (2)

| Page Break |  |
| --- | --- |

| 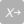 |
| --- |

Q4.16

You are doing a ward round and reviewing this patient. It is day 3 since admission, and for relevant cases, it is post-operative day 1.

**Scenario 16**

| Management/ operation | Adhesiolysis |
| --- | --- |
| Albumin, g/L | <25 |
| BMI, Kg/m^2^ | 18.5-24.9 |
| Unintentional weight loss, % in 3-6 months | 0-4.9 |
| Days without oral intake until now | 1-4 |
| Days likely to be without oral intake from now | ≥7 |

Would you start artificial nutritional support to meet the requirements of this patient? Select one answer.

- Yes (1)
- No (2)

| 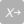 |
| --- |

Q4.17

You are doing a ward round and reviewing this patient. It is day 3 since admission, and for relevant cases, it is post-operative day 1.

**Scenario 17**

| Management/ operation | Adhesiolysis |
| --- | --- |
| Albumin, g/L | ≥35 |
| BMI, Kg/m^2^ | ≥30 |
| Unintentional weight loss, % in 3-6 months | >10 |
| Days without oral intake until now | 1-4 |
| Days likely to be without oral intake from now | 1-4 |

Would you start artificial nutritional support to meet the requirements of this patient? Select one answer.

- Yes (1)
- No (2)

| 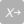 |
| --- |

Q4.18

You are doing a ward round and reviewing this patient. It is day 3 since admission, and for relevant cases, it is post-operative day 1.

**Scenario 18**

| Management/ operation | SB resection + anastomosis |
| --- | --- |
| Albumin, g/L | <25 |
| BMI, Kg/m^2^ | <18.5 |
| Unintentional weight loss, % in 3-6 months | >10 |
| Days without oral intake until now | ≥7 |
| Days likely to be without oral intake from now | 5-6 |

Would you start artificial nutritional support to meet the requirements of this patient? Select one answer.

- Yes (1)
- No (2)

| 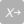 |
| --- |

Q4.19

You are doing a ward round and reviewing this patient. It is day 3 since admission, and for relevant cases, it is post-operative day 1.

**Scenario 19**

| Management/ operation | Continue conservative management |
| --- | --- |
| Albumin, g/L | <25 |
| BMI, Kg/m^2^ | 18.5-24.9 |
| Unintentional weight loss, % in 3-6 months | 5-10 |
| Days without oral intake until now | 1-4 |
| Days likely to be without oral intake from now | 5-6 |

Would you start artificial nutritional support to meet the requirements of this patient? Select one answer.

- Yes (1)
- No (2)

| 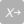 |
| --- |

Q4.20

You are doing a ward round and reviewing this patient. It is day 3 since admission, and for relevant cases, it is post-operative day 1.

**Scenario 20**

| Management/ operation | SB resection + stoma |
| --- | --- |
| Albumin, g/L | 30-34.9 |
| BMI, Kg/m^2^ | ≥30 |
| Unintentional weight loss, % in 3-6 months | 5-10 |
| Days without oral intake until now | 1-4 |
| Days likely to be without oral intake from now | 5-6 |

Would you start artificial nutritional support to meet the requirements of this patient? Select one answer.

- Yes (1)
- No (2)

| Page Break |  |
| --- | --- |

| 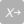 |
| --- |

Q4.21

You are doing a ward round and reviewing this patient. It is day 3 since admission, and for relevant cases, it is post-operative day 1.

**Scenario 21**

| Management/ operation | Continue conservative management |
| --- | --- |
| Albumin, g/L | <25 |
| BMI, Kg/m^2^ | 25-29.9 |
| Unintentional weight loss, % in 3-6 months | 5-10 |
| Days without oral intake until now | 1-4 |
| Days likely to be without oral intake from now | 1-4 |

Would you start artificial nutritional support to meet the requirements of this patient? Select one answer.

- Yes (1)
- No (2)

| 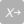 |  |
| --- | --- |

Q4.22

You are doing a ward round and reviewing this patient. It is day 3 since admission, and for relevant cases, it is post-operative day 1.

**Scenario 22**

| Management/ operation | Continue conservative management |
| --- | --- |
| Albumin, g/L | ≥35 |
| BMI, Kg/m^2^ | <18.5 |
| Unintentional weight loss, % in 3-6 months | 5-10 |
| Days without oral intake until now | 5-6 |
| Days likely to be without oral intake from now | ≥7 |

Would you start artificial nutritional support to meet the requirements of this patient? Select one answer.

- Yes (1)
- No (2)

| 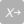 |
| --- |

Q4.23

You are doing a ward round and reviewing this patient. It is day 3 since admission, and for relevant cases, it is post-operative day 1.

**Scenario 23**

| Management/ operation | Continue conservative management |
| --- | --- |
| Albumin, g/L | 25-29.9 |
| BMI, Kg/m^2^ | ≥30 |
| Unintentional weight loss, % in 3-6 months | 5-10 |
| Days without oral intake until now | ≥7 |
| Days likely to be without oral intake from now | ≥7 |

Would you start artificial nutritional support to meet the requirements of this patient? Select one answer.

- Yes (1)
- No (2)

| 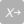 |
| --- |

Q4.24

You are doing a ward round and reviewing this patient. It is day 3 since admission, and for relevant cases, it is post-operative day 1.

**Scenario 24**

| Management/ operation | SB resection + stoma |
| --- | --- |
| Albumin, g/L | <25 |
| BMI, Kg/m^2^ | 25-29.9 |
| Unintentional weight loss, % in 3-6 months | >10 |
| Days without oral intake until now | 5-6 |
| Days likely to be without oral intake from now | ≥7 |

Would you start artificial nutritional support to meet the requirements of this patient? Select one answer.

- Yes (1)
- No (2)

| 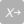 |
| --- |

Q4.25

You are doing a ward round and reviewing this patient. It is day 3 since admission, and for relevant cases, it is post-operative day 1.

**Scenario 25**

| Management/ operation | Adhesiolysis |
| --- | --- |
| Albumin, g/L | 25-29.9 |
| BMI, Kg/m^2^ | 25-29.9 |
| Unintentional weight loss, % in 3-6 months | 0-4.9 |
| Days without oral intake until now | 5-6 |
| Days likely to be without oral intake from now | 5-6 |

Would you start artificial nutritional support to meet the requirements of this patient? Select one answer.

- Yes (1)
- No (2)

End of Block: Scenarios

**Supplementary Figures and Tables**

**Table S1:** Preferences for nutritional support for each scenario according to surgeon seniority

| **Scenario** | **Consultants** | **Non-consultants** | **ST7-8/ post-CCT fellow** | **ST3-6** | **SAS doctor, associate specialist, trust grade** |
| --- | --- | --- | --- | --- | --- |
| 1 | 61.3 (46) | 60.3 (44) | 59.1 (13) | 62.2 (23) | 57.1 (8) |
| 2 | 96.0 (72) | 95.9 (70) | 100.0 (22) | 97.3 (36) | 85.7 (12) |
| 3 | 77.3 (58) | 72.6 (53) | 63.6 (14) | 75.7 (28) | 78.6 (11) |
| 4 | 73.3 (55) | 65.8 (48) | 59.1 (13) | 67.6 (25) | 71.4 (10) |
| 5 | 62.7 (47) | 54.8 (40) | 54.5 (12) | 54.1 (20) | 57.1 (8) |
| 6 | 93.3 (70) | 90.4 (66) | 86.4 (19) | 94.6 (35) | 85.7 (12) |
| 7 | 88.0 (66) | 84.9 (62) | 81.8 (18) | 86.5 (32) | 85.7 (12) |
| 8 | 70.7 (53) | 58.9 (43) | 63.6 (14) | 56.8 (21) | 57.1 (8) |
| 9 | 77.3 (58) | 75.3 (55) | 81.8 (18) | 78.4 (29) | 57.1 (8) |
| 10 | 100.0 (75) | 97.3 (71) | 95.5 (21) | 100.0 (37) | 92.9 (13) |
| 11 | 69.3 (52) | 54.8 (40) | 63.6 (14) | 51.4 (19) | 50.0 (7) |
| 12 | 76.0 (57) | 72.6 (53) | 77.3 (17) | 73.0 (27) | 64.3 (9) |
| 13 | 60.0 (45) | 46.6 (34) | 50.0 (11) | 43.2 (16) | 50.0 (7) |
| 14 | 90.7 (68) | 84.9 (62) | 90.9 (20) | 83.8 (31) | 78.6 (11) |
| 15 | 68.0 (51) | 46.6 (34) | 45.5 (10) | 43.2 (16) | 57.1 (8) |
| 16 | 92.0 (69) | 89.0 (65) | 86.4 (19) | 91.9 (34) | 85.7 (12) |
| 17 | 18.7 (14) | 11.0 (8) | 13.6 (3) | 10.8 (4) | 7.1 (1) |
| 18 | 100.0 (75) | 97.3 (71) | 100.0 (22) | 100.0 (37) | 85.7 (12) |
| 19 | 81.3 (61) | 75.3 (55) | 81.8 (18) | 75.7 (28) | 64.3 (9) |
| 20 | 58.7 (44) | 57.5 (42) | 68.2 (15) | 56.8 (21) | 42.9 (6) |
| 21 | 34.7 (26) | 23.3 (17) | 22.7 (5) | 18.9 (7) | 35.7 (5) |
| 22 | 98.7 (74) | 98.6 (72) | 100.0 (22) | 97.3 (36) | 100.0 (14) |
| 23 | 96.0 (72) | 100.0 (73) | 100.0 (22) | 100.0 (37) | 100.0 (14) |
| 24 | 100.0 (75) | 98.6 (72) | 100.0 (22) | 97.3 (36) | 100.0 (14) |
| 25 | 80.0 (60) | 82.2 (60) | 86.4 (19) | 81.1 (30) | 78.6 (11) |
| Overall preference | 77.7 (1443/1875) | 71.8 (1310/1825) | 73.3 (4.3/550) | 71.9 (665/925) | 69.1 (242/350) |

Consultant preference as reference value. Red = ≥5% below reference value; green ≥5% above reference value.

**Table S2:** Preferences for nutritional support for each scenario for consultants

| **Scenario** | **Work in IFU** | **Do not work in an IFU** |
| --- | --- | --- |
| 1 | 60.0 (6) | 61.5 (40) |
| 2 | 100.0 (10) | 95.4 (62) |
| 3 | 80.0 (8) | 76.9 (50) |
| 4 | 70.0 (7) | 73.8 (48) |
| 5 | 80.0 (8) | 60.0 (39) |
| 6 | 90.0 (9) | 93.8 (61) |
| 7 | 90.0 (9) | 87.7 (57) |
| 8 | 70.0 (7) | 70.8 (46) |
| 9 | 70.0 (7) | 78.5 (51) |
| 10 | 100.0 (10) | 100.0 (65) |
| 11 | 100.0 (10) | 64.6 (42) |
| 12 | 80.0 (8) | 75.4 (49) |
| 13 | 60.0 (6) | 60.0 (39) |
| 14 | 90.0 (9) | 90.8 (59) |
| 15 | 70.0 (7) | 67.7 (44) |
| 16 | 100.0 (10) | 90.8 (59) |
| 17 | 20.0 (2) | 18.5 (12) |
| 18 | 100.0 (10) | 100.0 (65) |
| 19 | 90.0 (9) | 80.0 (52) |
| 20 | 70.0 (7) | 56.9 (37) |
| 21 | 50.0 (5) | 32.3 (21) |
| 22 | 100.0 (10) | 98.5 (64) |
| 23 | 100.0 (10) | 95.4 (62) |
| 24 | 100.0 (10) | 100.0 (65) |
| 25 | 80.0 (8) | 80.0 (52) |
| Overall preference | 80.8 (202/250) | 76.4 (1241/1625) |

Work in IFU preference as reference value. Red = ≥5% below reference value; green ≥5% above reference value.

**Table S3:** Preferences for nutritional support for each scenario according to experience of intestinal failure

| **Scenario** | **IF experience** | **No IF experience** |
| --- | --- | --- |
| 1 | 60.9 (42) | 60.8 (48) |
| 2 | 98.6 (68) | 93.7 (74) |
| 3 | 72.5 (50) | 77.2 (61) |
| 4 | 66.7 (46) | 72.2 (57) |
| 5 | 66.7 (46) | 51.9 (41) |
| 6 | 94.2 (65) | 89.9 (71) |
| 7 | 85.5 (59) | 87.3 (69) |
| 8 | 62.3 (43) | 67.1 (53) |
| 9 | 76.8 (53) | 75.9 (60) |
| 10 | 98.6 (68) | 98.7 (78) |
| 11 | 65.2 (45) | 59.5 (47) |
| 12 | 75.4 (52) | 73.4 (58) |
| 13 | 58.0 (40) | 49.4 (39) |
| 14 | 87.0 (60) | 88.6 (70) |
| 15 | 60.9 (42) | 54.4 (43) |
| 16 | 92.8 (64) | 88.6 (70) |
| 17 | 15.9 (11) | 13.9 (11) |
| 18 | 98.6 (68) | 98.7 (78) |
| 19 | 81.2 (56) | 75.9 (60) |
| 20 | 62.3 (43) | 54.4 (43) |
| 21 | 33.3 (23) | 25.3 (20) |
| 22 | 100.0 (69) | 97.5 (77) |
| 23 | 98.6 (68) | 97.5 (77) |
| 24 | 100.0 (69) | 98.7 (78) |
| 25 | 79.7 (55) | 82.3 (65) |
| Overall preference | 75.7 (1305/1725) | 73.3 (1448/1975) |

IF experience preference as reference value. Red = ≥5% below reference value; green ≥5% above reference value.
